# Oncogene-induced NAD^+^ depletion in tumorigenesis

**DOI:** 10.18632/oncoscience.154

**Published:** 2015-04-19

**Authors:** Krishna S. Tummala, Nabil Djouder

**Affiliations:** Cancer Cell Biology Programme, Growth Factors, Nutrients and Cancer Group, Spanish National Cancer Research Centre, CNIO, Madrid, Spain

A proto-oncogene (hereafter simplified as oncogene) has the potential to initiate cancer. Oncogenes are often mutated and/or their products are expressed at high levels in various types of cancer. Several mechanisms and features of oncogenesis have been extensively discussed in the review of Hanahan and Weinberg “hallmarks of cancer: the next generation” [1]. Fundamentally, oncogenes give an unlimited proliferative advantage to cells which generates replicative stress and, leads to the genetic heterogeneity of the cell population. Thus, cancer is the disease of the genome and, targeting the terminal phase often results in cancer recurrence.

Several ongoing preclinical studies and clinical trials propose to target the proliferation process [2,3]. Specific inhibitors of the replicative stress were successfully validated for cancer treatment in mice [4]. This type of therapy clearly induces cancer cell death but unfortunately creates an irreversible genotoxic stress in healthy cells, which may lead to their transformation, consequent cellular dysfunctions and secondary cancer. There is a clear need of developing new therapies. Understanding the critical early event and dissecting the step-wise progression of tumorigenesis would help us to design more efficient therapeutic interventions to prevent and treat cancer. In particular because, metabolic alterations are common fundamental characteristics of oncogenes and in this context, represent an essential hallmark of cancer, tackling primary metabolic defects can be an elegant approach to prevent and cure cancer. We summarize in this short editorial a recent example reported in Tummala et al. of how targeting the metabolic defects prior to the high cellular proliferation and DNA damage can eradicate hepatocellular carcinoma (HCC) and prevent pancreatic cancer development [5].

HCC is the most frequent primary liver neoplasm which often arises in the predisposing liver disease states. HCC accounts for approximately 800,000 deaths each year and, making it the second most lethal cause of cancer worldwide (GLOBOCAN 2008 v2.0). Various therapeutic approaches to the treatment of advanced HCC have been unsuccessfully implemented. One of the most beneficial HCC treatment is so far the well known kinase inhibitor Sorafenib that improves patient survival of a maximum of 2 to 3 months [2,6]. Thus, limited and inefficient therapeutic options render the curative treatment of the disease almost impossible.

Although several pathways and molecular players were reported in HCC development, the lack of animal models that recapitulate the full spectrum of the human disease progression may impede the development of suitable therapies. Despite detailed etiological and clinical features, the pathogenesis of HCC is not well understood. The comprehension of the disease, identifying clinically relevant therapeutic targets and the generation of efficient medicines require powerful genetic tools that mimic the human clinical stages. In a recent study of our lab, we generated genetically engineered mouse models (GEMMs) of Unconventional prefoldin RPB5 interactor (URI) loss- and gain-of-function [5,7]. Development of tumors in the murine liver after ectopic URI expression in the whole body motivated us to study its role and function in liver disease. Hepatocytic specific URI expression leads to spontaneous, heterogenous and aggressive tumors after 65 weeks of age, through a multistep process that recapitulate the human features of HCC. We propose that URI is as an oncogene essential for liver tumorigenesis and, URI GEMMs represent unique genetic models to appropriately address the mechanisms of HCC development and explore novel therapeutic avenues.

**Figure 1:**
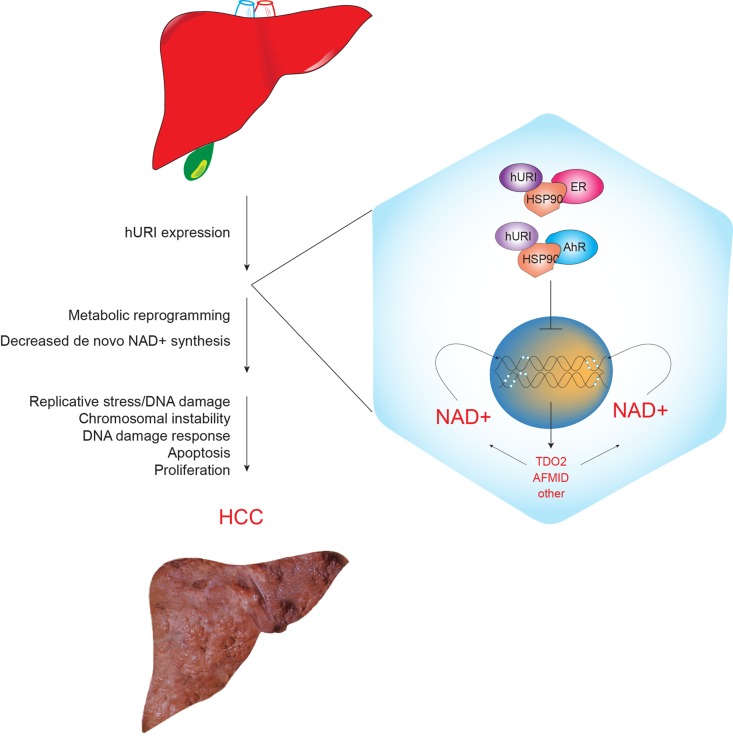
Scheme representing hepatocyte specific hURI expression leading to DNA damage and liver tumorigensesis Mechanistically, we demonstrate that URI inhibits de novo NAD^+^ synthesis through cytoplasmic sequestration of aryl hydrocarbon and estrogen receptors (AhR and ER, respectively), both of them are transcription factors of enzymes implicated in catabolism of tryptophan to NAD^+^ synthesis [5].

At the early stages, we demonstrate that DNA damage is the critical initiating event leading to dysplastic lesions and aggressive HCC. Interestingly, while apoptosis-induced compensatory proliferation is suggested to initiate liver tumors, in our model, abolishing apoptosis and increasing genotoxic stress by inactivating p53, accelerates tumor formation and death of mice. Thus, oncogenic URI-induced genotoxic stress, rather than excessive liver injury is essential to initiate the liver tumorigenic process. In support of this, chromosomal abnormalities represent the most reliable clinical aspect to determine precancerous stages of HCC [8].

Next, using global quantitative transcriptomic and proteomic analysis, we demonstrate that prior to DNA damage, URI downregulates the L-tryptophan/kynurenine catabolism pathway and thus, leads to the inhibition of de novo NAD^+^ synthesis. The decrease in total NAD^+^ levels consequently provokes DNA damage (Figure). Although it remains unclear how decreased in NAD^+^ concentrations causes genotoxic stress, preliminary results indicate that the DNA repair protein poly-ADP-ribose polymerase (PARP) activity may be affected. We do not completely exclude that NAD^+^ depletion may affect Sirts activity. Furthermore, because NAD^+^ is a cofactor for inosine monophosphate dehydrogenase implicated in dNTPs synthesis, NAD^+^ deficits can also lead to insufficient dNTP production that may contribute to DNA damage during high replication. Replenishing the NAD^+^ levels by nicotinamide riboside (NR), a derivative of vitamin B3, prevented and abolished DNA damage and aggressive tumor formation [5].

Based on previous observations and extending our work to other oncogenes known to induce tumors on the basis of DNA damage, we demonstrate that c-Myc expression in pancreas induced NAD^+^ depletion through a net reduction in tryptophan catabolism. Enzymes implicated in tryptophan degradation are downregulated by c-Myc over-expression. NAD^+^ depletion is apparently involved in the formation of pancreatic tumors. Importantly, these tumors could be tackled when NAD^+^ levels were enhanced by NR supplementation [5]. We propose that NAD^+^ depletion is a common molecular mechanistic basis for oncogene-induced DNA damage and tumor development.

NR seems therefore to be an efficient therapy for the treatment of various cancers in which predictive and prognostic factors can be identified as oncogene-associated genotoxic stress. Clinical trials with NR for treatment of such cancers are under consideration. However, developing a methodological screen to find more efficient and stable NAD^+^ “boosters” and, understanding the mechanisms of NAD^+^ depletion-dependent DNA damage would offer a broad spectrum of new possibilities to decisively prevent and cure cancer in human beings. The development of drug discovery platform based on screening of new compounds that enable to abolish DNA damage by increasing NAD^+^ levels can thus be an exciting investment in the war against cancer.

## References

[1] Hanahan D, Weinberg RA (2011). Cell.

[2] El-Serag HB (2011). N. Engl. J. Med..

[3] Llovet JM (2005). J. Gastroenterol..

[4] Toledo LI (2011). Mol. Oncol..

[5] Tummala KS (2014). Cancer Cell.

[6] Llovet JM (2008). N. Engl. J. Med..

[7] Djouder N (2007). Mol. Cell..

[8] Kudo M (2009). J. Gastroenterol..

